# Parameters that remain consistent independent of pausing before gait-initiation during normal rise-to-walk behaviour delineated by sit-to-walk and sit-to-stand-and-walk

**DOI:** 10.1371/journal.pone.0205346

**Published:** 2018-10-09

**Authors:** Gareth D. Jones, Darren C. James, Michael Thacker, David A. Green

**Affiliations:** 1 Centre for Human and Applied Physiological Sciences (CHAPS), King's College London, London, United Kingdom; 2 Physiotherapy Department, Guy’s & St. Thomas’ NHS Foundation Trust, London, United Kingdom; 3 Sport and Exercise Science Research Centre, London South Bank University, London, United Kingdom; University of Illinois, UNITED STATES

## Abstract

**Background:**

Rising-to-walk is an everyday transitional movement task rarely employed in gait rehabilitation. Sit-to-walk (STW) and sit-to-stand-and-walk (STSW), where a pause separates sit-to-stand and gait-initiation (GI) represent extremes of rising-to-walk behaviour. Delayed GI can indicate pathological impairment but is also observed in healthy individuals. We hypothesise that healthy subjects express consistent biomechanical parameters, among others that differ, during successful rising-to-walk task performance regardless of behaviour. This study therefore sought to identify if any parameters are consistent between STW and STSW in health because they represent normal rise-to-walk performance independent of pause, and also because they represent candidate parameters sensitive enough to monitor change in pathology.

**Methods:**

Ten healthy volunteers performed 5 trials of STW and STSW. Event timing, ground-reaction-forces (GRFs), whole-body-centre-of-mass (BCoM) displacement, and centre-of-pressure (CoP) to extrapolated BCoM (xCoM) distance (indicator of positional stability) up to the 3^rd^ step were compared between-tasks with paired *t*-tests. For consistent parameters; agreement between-tasks was assessed using Bland-Altman analyses and minimal-detectable-change (MDC) calculations.

**Results:**

Mean vertical GRFs, peak forward momentum and fluidity during rising; CoP-xCoM separation at seat-off, upright, GI-onset, and steps1-2; and forward BCoM velocity were all significantly greater in STW. In contrast, peak BCoM vertical momentum, flexion-momentum time, and 3^rd^ step stability were consistent between tasks and yielded acceptable reliability.

**Conclusion:**

STW is a more challenging task due to the merging of rising with GI reflected by greater CoP-xCoM separation compared to STSW indicative of more positional instability. However, BCoM vertical momentum, flexion-momentum time, and step3 stability remained consistent in healthy individuals and are therefore candidates with which to monitor change in gait rehabilitation following pathology. Future studies should impose typical pause-durations observed in pathology upon healthy subjects to determine if the parameters we have identified remain consistent.

## Introduction

Humans often transition between postures as part of daily life. For example working adults are reported to rise from a seated position more than 60 times per day [1] and healthy individuals have been found to initiate walking from sedentary positions including siting over 90% of the time (rising-to-walk) [2]. Whilst ubiquitous, rising-to-walk is also a flexible transitional task. It can be undertaken smoothly, as in sit-to-walk (STW) where sit-to-stand (STS) is integrated fluidly with gait-initiation (GI) [2]. Yet it can equally be executed with increasing time between STS and GI up to where they are separated [3] as part of a normal dual task; for example when a seated individual rises but pauses to check their pockets before they set off walking.

In contrast to healthy individuals’ rise-to-walk flexibility, pathology results in inflexibility manifested by separation of STS and GI when STW is attempted—as observed in Parkinson’s disease [4], stroke [5], and aging [6]. Furthermore, in the clinical practice of gait-rehabilitation, a pause is often encouraged before GI is attempted after rising—termed sit-to-stand-and-walk (STSW). Indeed, STW is rarely employed during acute rehabilitation with STSW more commonly employed, presumably due to lower task complexity [7], and thus perceived risk. Consequently, it is rarely considered whether a patient can attempt STW rather than STSW, despite the possibility that STW might confer a more challenging, and thus effective adaptation stimulus [8]. With rising-to-walk fluidity limited by pathology, and modulated dependent on the movement context by healthy individuals there is a resultant spectrum of rising-to-walk behaviour delineated by the extremes of STW and STSW.

An indicator of this spectrum is the hesitation index (HI) [9, 10]. STW is characterised by a signature depression in whole-body-centre-of-mass (BCoM) horizontal momentum immediately after seat-off, and the HI describes the magnitude of the depression expressed as a proportion of maximum momentum during rising. A low HI indicates superior fluidity as seen in healthy individuals [11] who tend to task-consistency by controlling for the abundant degrees of freedom (DOF) of the effector system–healthy individuals are able to utilise available DOFs in the face of perturbations and the resultant low HIs are therefore a function of healthy individuals’ motor abundance [12]. In contrast, HIs ≥50% indicate lack of movement control flexibility and have been observed in older adults at risk of falling [2]. The ability of the HI to discriminate between health and pathology when individuals perform STW to the best of their ability is encouraging, even though distinct cut-offs are yet to be determined [2]. Furthermore, a floor effect resulting from its expression as a percentage renders it unable to distinguish behaviour differences once HIs approach 100%.

Similarly, temporal parameters can be used to monitor rise-to-walk behaviour, such as the length of time between STS and GI, or pause-time, defined as the interval between reaching upright and GI-onset. However, this definition cannot describe pause durations across the rise-to-walk spectrum because in STW, GI-onset precedes reaching upright. An alternative is to use the interval between seat-off and GI-onset, or the transition phase [5]. Similarly to the HI however, the transition phase duration is unable to wholly discriminate between healthy and pathological rise-to-walk behaviour when healthy individuals can adapt their behaviour while maintaining rise-to-walk task success across the entire spectrum of transition phase durations.

An alternative approach would be to determine parameters that are consistent, irrespective of whether STW or STSW are performed. We hypothesise that consistent biomechanical parameters exist across the spectrum of rise-to-walk behaviour (STW and STSW) independent of pause in healthy individuals. The identification of any consistent parameters in health could represent candidate parameters sensitive enough to monitor change in pathology, thereby facilitating characterisation of rising-to-walk performance during rehabilitation.

Thus in the present study, we tested healthy participants undertaking a low risk rising-to-walk protocol, suitable for neurologically impaired patients [13], leading with their non-dominant limb (analogous to an affected-limb in stroke [5]). In order to ensure our low risk protocol did not introduce task resemblance, we aimed to confirm expected biomechanical differences between STW and STSW reflective of self-selected pausing. Our primary aim however was the identification of consistent parameters that may represent rise-to-walk performance independent of pause.

## Methods

The London South Bank University Ethics Committee approved this study (UREC1413/2014). Participants gave written informed consent before data collection began.

### Participants

Ten healthy volunteers (5F, 5M; Mean (±SD): 29.1±7.7years, 171.0±7.7cm, 73.5±10.9kgm, knee-height (KH) 461±37mm, bi-acromial (shoulder) width 407±42mm) provided written informed consent to participate in this local ethical committee approved study (UREC1413/2014).

### Experimental procedure

**Fig 1:**
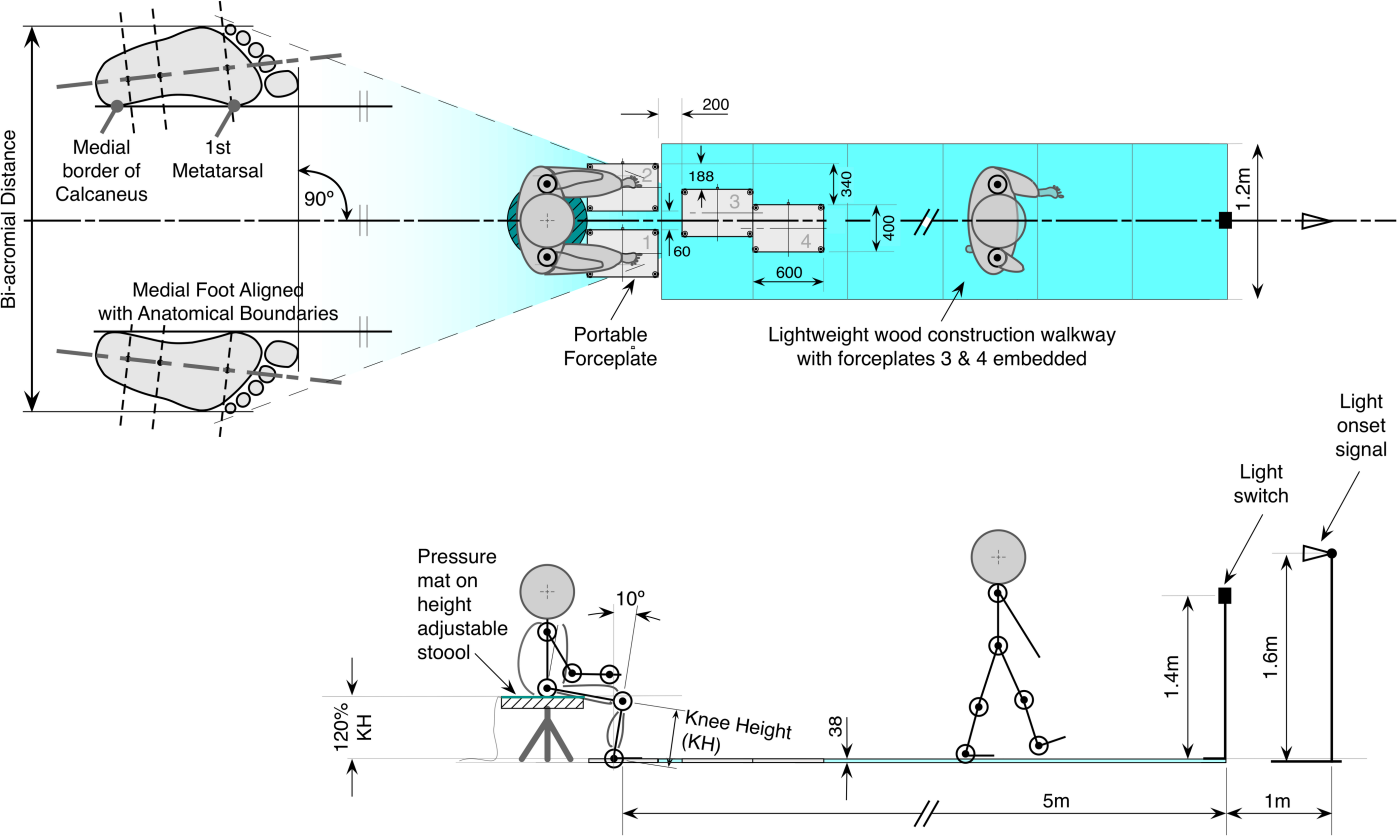
Experimental setup. This example shows a left-leg lead configuration: Participants sat on an instrumented stool at 120% knee height (KH), with ankles 10° in dorsiflexion, and feet at shoulder width apart orientated forward. In both STW and STSW task conditions on a light onset cue, participants rose with their feet on independent force plates, walked forward 5m over two further force plates embedded in the walkway with their arms unconstrained, and stopped to turn off the light at a switch. Participants performed 5 trials of each task leading with their non-dominant limb at self-selected pace. All dimensions in mm unless otherwise stated. Not to scale.

Participants attended the gait laboratory once, and upon a visual cue after which they were instructed to move when ready, performed 10 rise-to-walk trials (5 STW and STSW trials) in a randomised order (at self-selected speed) leading with their non-dominant limb. Participants rose from an instrumented (pressure-mat, Arun Electronics Ltd, UK) height-adjustable stool (Svenerik, Ikea, Sweden) set at 120%KH (floor to dominant knee joint-line distance), with feet at bi-acromial distance and 10° of ankle dorsiflexion (Fig 1) [14]. Participants walked forward 5m, stopped, and turned off the light at a switch to end the trial. In STW, participants were instructed to rise and immediately walk forwards once the light signal was operated, whereas in STSW they were instructed to stop and pause upon standing. Pause duration was self-selected within the context of an instruction to mentally count from 1–3 before walking. In both conditions, subjects’ arms were unconstrained. They were instructed to place their arms in front of them with hands above their thighs while they waited for the light signal in order to reduce marker obstruction. However, they were instructed to use their arms naturally once they decided to rise from the seated position.

The whole body was modelled as 13 rigid segments (feet, shanks, thighs, pelvis, torso, upper-arms, forearms, head) and was reconstructed by tracking trajectories of 47 reflective markers (Qualysis, Medical AB, Gothenburg, Sweden) placed onto the skin overlying anatomical landmarks, and attached to rigid bases. Kinematic data were acquired using an eight-camera optical motion analysis system (Oqus 3-series, Qualisys Medical AB, Gothenburg, Sweden) sampled at 60Hz and synchronised (1020Hz) with analogue data from 4 force plates (FPs) width 400mm, length 600mm mounted within the 5m walkway (9281E, Kistler Instruments AG, Switzerland), the stool pressure-mat, and light-switch. Two FPs were located under each foot to capture ground reaction forces (GRFs) during rising (Fig 1), with two more positioned to capture GRFs up to step3 [14]. In the event participants did not interface with individual force plates cleanly, the trial was repeated.

### Data processing

Raw marker trajectories and GRF data were exported into Visual 3D software (C-Motion Inc., Germantown, USA) and smoothed with 10Hz and 25Hz 4^th^ order low-pass Butterworth filters respectively [14]. In order to establish step changes in light and pressure-mat analogue voltage signals necessary to determine light-on and seat-off events, they were filtered by 25-point window averaging.

### Data analysis

Data between movement-onset and 3^rd^ initial-contact were used for analysis. Movement parameters (Table 1) were delineated with respect to task phases (Table 2) [5, 6, 9, 10, 15]. The two dimensional xCoM position was calculated based on Hof [16], with inverted pendulum length defined as the BCoM vertical height [17].

**Table 1:** Definintion of movement events during STW and STSW.

Movement Event	Definition		Movement Parameter
Light-On	Instance determined as the point when the light analogue channel voltage drops below mean-3SDs voltage for >8 frames (133ms) of 1s quiet sitting		–
Movement-Onset	Instance determined when BCoM forward velocity increases for >8 frames (133ms) beyond the mean+3SD BCoM vertical velocity during 1s of quiet sitting displacement before light-on		Response Phase-Time (s)
Peak BCoM Momenta	Instances of first peak anteroposterior, mediolateral and vertical BCoM momentum signal occurring before upright event		Peak momenta (kg.m.s^-1^) (x, y, z)
Seat-Off	Instance determined as the point at which the seat-mat analogue channel voltage drops below the mean-3SD baseline voltage for >8 frames (133ms) of 1s quiet sitting		Flexion Momentum Phase-Time, CoP-xCoM Dist (m)
Peak Arm Segment Momenta	Instances of peak anteroposterior, mediolateral and vertical dominant-arm momentum signal occurring before upright event		Peak arm momenta (kg.m.s^-1^) (x, y, z)
Peak GRF	Instance of peak summated force plates 1 and 2 (and swing limb force plate) anteroposterior, mediolateral and vertical GRF signals occurring between movement onset and seat-off events		Peak summated GRF (%BW) (x, y, z), Peak swing limb GRF (%BW) (x, y, z)
Minimum anteroposterior BCoM Momentum	First minima in BCoM anteroposterior momentum after Peak BCoM Momentum event (the HI is expressed as the percentage of the minima with respect to the first peak BCoM anteroposterior momentum preceding it)		HI (%)
Upright	Instance of initial peak vertical (z-component) BCoM displacement signal occurring between seat-off and first initial contact events		Rising Phase-Time (s), CoP-xCoM Dist (m)
	STW	STSW	
GI Onset	Instance of peak swing limb force plate vertical (z-component) GRF signal occurring between movement onset and HO1 events	First instance when CoP lateral velocity signal breaches 0.0m/s threshold for > 8 frames (133ms) occurring between Upright and HO1 events	Transition Phase-Time (s), Stance BOS
1^st^ Heel-Off (HO1)	Instance when swing lib calcaneal marker vertical (z-component) velocity breaches 0.0m/s threshold for > 8 frames (133ms)		–
1^st^ Toe-Off (TO1)	Instance when swing limb force plate vertical (z-component) GRF signal drops <20N for >8 frames (133ms) occurring after Seat-Off event		GI Phase-Time, GI Velocity (m.s-^1^), CoP-xCoM Dist (m)
1^st^ Initial Contact (IC1)	Instance when force plate 3 vertical (z-component) GRF signal increases >20N for >8 frames (133ms) occurring after TO1 event		Step 1 Phase-Time (s) & Velocity (m.s^-1^), Step 1 max CoP-xCoM Dist (m)
2^nd^ Initial Contact (IC2)	Instance when force plate 4 vertical (z-component) GRF signal increases >20N for >8 frames (133ms) occurring after IC1 event		Step 2 Phase-Time (s) & Velocity (m.s^-1^), Step 2 max CoP-xCoM Dist (m)
3^rd^ Initial Contact (IC3)	Instance when initial swing limb calcaneus marker (CALC) vertical velocity breaches threshold of 0.0m/s for >8 frames (133ms) occurring after IC2 event		Step 3 Phase-Time (s) & Velocity (m.s^-1^), Step 3 max CoP-xCoM Dist (m), Total Movement Time (s)
Base of Support (BOS)	Horizontal distance between calcanei, accounting for marker diameter, perpendicular to the anteroposterior global coordinate system axis		Width at Step 1, 2, 3

**Table 2:** Definition of movement phases during STW and STSW.

	Movement Phase									
	Response	Flexion Momentum	Rising	Transition	Gait Initiation	Step 1	Step 2	Step 3	Stepping	Movement
Start	Light-On	Movement-Onset	Movement-Onset	Seat-Off	GI-Onset	1^st^ Toe-Off (TO1)	1^st^ Initial-Contact (IC1)	2^nd^ Initial-Contact (IC2)	1^st^ Toe-Off (TO1)	Movement-Onset
End	Movement-Onset	Seat-Off	Upright	GI-Onset	1^st^ Toe-Off (TO1)	1^st^ Initial-Contact (IC1)	2^nd^ Initial-Contact (IC2)	3^rd^ Initial-Contact (IC3)	3^rd^ Initial-Contact (IC3)	3^rd^ Initial-Contact (IC3)
Features	Postural preparation activity undertaken in response to light signal before movement onset	Forward flexion movement of trunk. Vertical force drops before increasing rapidly. Forward BCoM velocity increases	Forward flexion movement of trunk. Extension of lower limb joints and elevation of head, arm, trunk segments before BCoM reaches its peak initial vertical displacement	Period between forward flexion movement of trunk, and task-specific onset of gait initiation	Initiation of anticipatory postural adjustment when vertical projection of CoP and BCoM separate, or pre-loading of swing limb near Seat-Off, followed by rapid unloading & swing limb. Toe-Off	First single support phase, forward BCoM acceleration	First between-limb event cycle, forward BCoM acceleration	Second between-limb event cycle, if gait continues then steady-state average BCoM velocity achieved here	Whole stepping time	Whole movement time

BCoM–whole body centre-of-mass; BOS–base of support; BW–body weight; CoP–centre-of-pressure; GI–gait initiation; GRF–ground reaction force; HI–hesitation index;

SD–standard deviation

All data were normally distributed (Kolmogorov-Smirnov test), therefore the effect of task on movement parameters was determined via paired-sample *t*-tests, using individual mean data derived from 5 trials in each condition (expressed as mean ±SD). Cohen’s *d* was used to indicate effect size and 95% confidence intervals (95%CI) were used to indicate how sample means relate to the population.

Parameters where no significant task-effect was found were considered consistent but were subjected to further assessment of their between-task agreement and intra-subject reliability. Between-task agreement was assessed using Bland-Altman analyses of repeated-measures [18], with the true value assumed to be constant [19] in calculations of 95% limits of agreement (LOA) and their 95%CI. Systematic bias was evaluated using 1-sample *t*-tests to assess variation of between-task differences around zero. Proportional bias (heteroscedasticity) was evaluated using linear regression with R^2^ values used to report the percentage of variance in the dependent variable (between-task difference) explaining the independent variable (between-task average).

Intra-subject reliability of consistent parameters was assessed for one task (STW) since there would be no significant difference between STW and STSW in these parameters. Two-way random effect model intra-class correlation coefficients (ICC_2,1_) where >0.75 are deemed acceptable was used [20]. The ICCs were used to calculate standard error of measurement (SEM), minimal detectable change (MDC) and %MDC. These parameters represent; measurement error, the minimum amount of difference between two measurements below which there is more than a 95% chance that no real difference exists, and the proportional size of that difference with respect to the mean of all observations from STW and STSW, respectively [21]. The full dataset is available from the Dryad Digital Repository at https://doi.org/10.5061/dryad.bv3c8b5.

## Results

### Differences between-tasks

#### Response, rising, transition, and GI Phases

There was no difference in mean (SD) [95%CI] response phase-time (STW: 0.30s (0.07) [0.25–0.36]; STSW 0.32s (0.08) [0.26–0.38]) and stance width at GI-onset (STW: 0.27m (0.04) [0.24–0.30]; STSW: 0.27m (0.04) [0.24–0.29]) between tasks. During the transition phase, peak net GRFs were significantly greater in both medio-lateral (towards the stance-limb) [d = 3.091, *p*<0.001] and vertical [*d* = 0.767, *p* = 0.038] directions during STW (Table 3).

**Table 3:** Mean (SD) [95%CI] for movement parameters with significant difference between STW and STSW.

Phase	Movement Parameter	STW			STSW			
		Mean	(SD)	[95%CI]	Mean	(SD)	[95%CI]	p
Transition	Peak Net Medio-lateral† GRF (Body Weight %)	6.74	(1.26)	[5.84–7.64]	1.65	(0.57)	[1.24–2.05]	<0.001
	Peak Net Anteroposterior‡ GRF (Body Weight %)	-6.33	(1.80)	[-7.62–5.04]	-9.71	(2.16)	[-11.26–8.17]	<0.001
	Peak Net Vertical GRF (Body Weight %)	127.73	(6.72)	[122.93–132.54]	123.36	(7.79)	[117.78–128.93]	0.038
	Peak Dominant-Arm Medio-lateral† Momentum (kg.m.s^-1^)	0.39	(0.13)	[0.30–0.49]	0.26	(0.10)	[0.19–0.34]	<0.001
	Peak Dominant-Arm Anteroposterior‡ Momentum (kg.m.s^-1^)	2.60	(0.46)	[2.27–2.93]	2.19	(0.31)	[1.97–2.42]	<0.001
	Peak Dominant-Arm Vertical Momentum (kg.m.s^-1^)	1.35	(0.38)	[1.08–1.62]	2.37	(0.71)	[1.86–2.88]	<0.001
GI	GI Phase-Time (GI-Onset » TO1) (s)	0.34	(0.08)	[0.29–0.40]	0.63	(0.08)	[0.57–0.69]	<0.001
	GI BCoM Forward Velocity (m.s^-1^)	0.45	(0.10)	[0.38–0.53]	0.10	(0.04)	[0.08–0.13]	<0.001
	Peak Swing Limb Medio-lateral† GRF (Body Weight %)	7.76	(1.97)	[6.36–9.17]	9.91	(1.62)	[8.75–11.07]	0.005
	Peak Swing Limb Anteroposterior‡ GRF (Body Weight %)	3.35	(0.76)	[2.81–3.90]	5.85	(2.48)	[4.08–7.63]	0.006
	Peak Swing Limb Vertical GRF (Body Weight %)	77.78	(7.63)	[72.32–83.24]	72.13	(6.21)	[67.69–76.57]	0.044
Stepping	Step 1-Step 3 BCoM Forward Velocity (m.s^-1^)	1.33	(0.18)	[1.20–1.45]	1.23	(0.15)	[1.12–1.33]	<0.001
All	Overall Movement Time (Movt-Onset » IC3) (s)	2.59	(0.24)	[2.42–2.75]	4.45	(0.64)	[3.99–4.91]	<0.001

†Absolute values given;

‡Positive values indicate anterior direction; BCoM–whole-body-centre-of-mass;

GI–gait initiation; GRF–ground reaction force; IC3 – 3rd initial contact; TO1 – 1st toe-off

However, STW peak net GRFs were significantly lower [*d* = 1.989, *p*<0.001] in the posterior direction [*d* = 1.989, *p*<0.001]. The contribution of dominant-arm momentum during transition was greater during STW in both the medio-lateral (away from the body) [*d* = 1.725, *p*<0.001] and anterior directions [*d* = 1.835, *p*<0.001], but was significantly lower [*d* = 2.047, *p*<0.001] vertically. Gait-initiation phase times were shorter [*d* = 2.171, *p*<0.001] with higher GI BCoM forward peak velocity [*d* = 4.103, *p*<0.001] in STW. During GI however, STW yielded lower swing-limb peak GRFs medio-laterally (towards the stance-limb) [*d* = 1.183, *p* = 0.005] and anteriorly [*d* = 1.116, *p* = 0.006] but not vertically, which was significantly greater [*d* = 0.741, *p* = 0.044] compared to STSW. Unsurprisingly, overall movement time was shorter in STW [*d* = 3.915, *p*<0.001] due to pause imposition (mean duration 0.84s (0.41) [0.55–1.13]) in STSW (Table 3).

**Fig 2:**
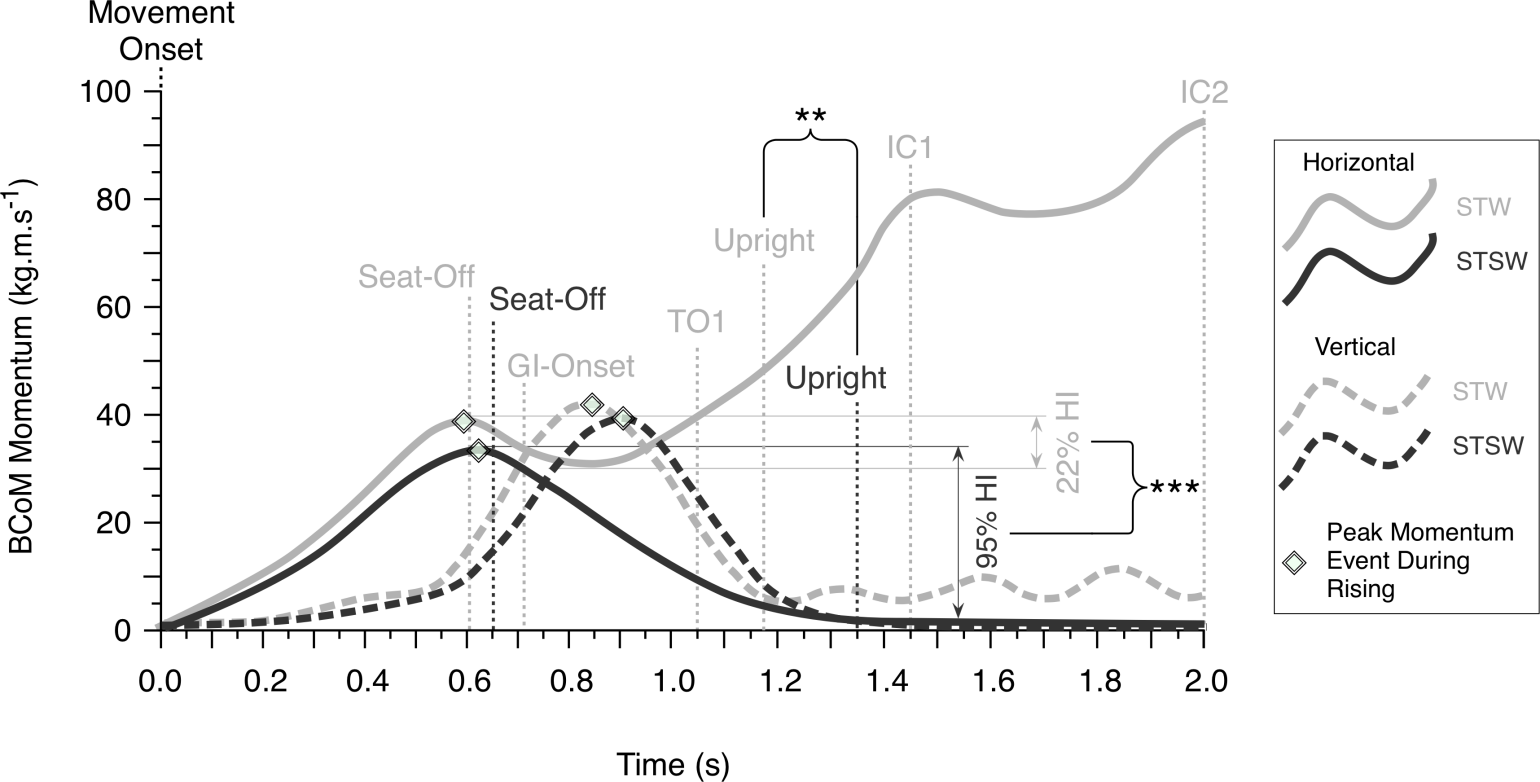
Mean STW and STSW horizontal and vertical mean BCoM momentum profiles. Group ensemble mean horizontal (solid lines) and vertical (dashed lines) momentum for STW (light shade) and STSW (dark shade). Vertical lines indicate mean time of movement events (Seat-Off, Gait-Initiation Onset (GI-Onset), 1st Toe-Off (TO1), Upright, 1st Initial-Contact (IC1) and 2nd Initial-Contact (IC2) with Movement-Onset representing the origin of x-axis). Peak momentum events, and mean Hesitation Indices (HI) during rising are indicated separately. Note that the time frame presented does not include GI-onset in STSW. **Statistically significant between tasks at <0.01; *** <0.001.

Mean HI in STW (22.42% (16.65) [10.51–34.33]) was substantially lower than STSW (95.08% (2.96) [92.96–87.19]) [*d* = 4.162, *p*<0.001]. The GI phase (GI-onset to 1^st^ toe-off (TO1)) was completed before upright was reached in STW where a more rapid rise time (movement-onset to upright) was observed in STW (1.17s (0.18) [1.04–1.30]) compared to STSW (1.35s (0.28) [1.15–1.56]) [*d* = 1.167, *p<*0.001] (Fig 2).

CoP-xCoM horizontal distances at seat-off [*d* = 1.824, *p*<0.001], upright [*d* = 5.971, *p*<0.001], GI-onset [*d* = 1.755, *p*<0.001], and TO1 [*d* = 0.786, *p* = 0.035] were all significantly greater in STW (Table 4).

**Table 4:** Mean (SD) [95%CI] comparison for CoP-xCoM horizontal distance at key movement events between STW and STSW.

		STW			STSW			
Movement Parameter		Mean	(SD)	[95%CI]	Mean	(SD)	[95%CI]	p
CoP-xCoM Distance (m)	Seat-Off	0.08	(0.03)	[0.07–0.10]	0.04	(0.01)	[0.03–0.05]	<0.001
	Upright	0.32	(0.04)	[0.28–0.35]	0.02	(0.01)	[0.02–0.03]	<0.001
	GI-Onset	0.11	(0.04)	[0.08–0.14]	0.03	(0.01)	[0.02–0.03]	<0.001
	TO1	0.22	(0.04)	[0.08–0.14]	0.20	(0.01)	[0.16–0.25]	0.035

CoP–centre-of-pressure; GI–gait initiation; TO1 – 1st toe-off; xCoM–extrapolated whole-body-centre-of-mass;

#### Steps 1–3

Mean BCoM forward velocity was significantly greater during step1 [*d* = 2.869, *p*<0.001], step2 [*d* = 1.404, *p* = 0.002], and step3 [*d* = 0.876, *p* = 0.022] in STW, although the difference between conditions diminished with each step. Additionally, significantly shorter stepping times were observed in STW for step1 [*d* = 0.0862, *p* = 0.023], step2 [*d* = 1.544, p = 0.001] and step3 [*d* = 1.002, *p* = 0.011]. There were no differences in foot width during step1 and 2, although in step3 a small but statistically significantly wider width was adopted in STW [*d* = 1.256, *p* = 0.003] (Table 5).

**Table 5:** Mean (SD) [95%CI] comparison for BCoM forward velocity, stepping time, and step width between STW and STSW.

Movement Parameter		STW			STSW			p
		Mean	(SD)	[95%CI]	Mean	(SD)	[95%CI]	
Step 1	Average Forward Velocity (m.s.^-1^)	0.71	(0.11)	[0.63–0.79]	0.57	(0.08)	[0.51–0.63]	<0.001
	Time (s)	0.41	(0.05)	[0. 34–0.44]	0.43	(0.06)	[0.39–0.48]	0.023
	Width	0.18	(0.04)	[0.16–0.21]	0.19	(0.04)	[0.16–0.21]	0.493
Step 2	Average Forward Velocity (m.s.^-1^)	1.11	(0.13)	[1.02–1.20]	1.03	(0.11)	[0.96–1.11]	0.002
	Time (s)	0.58	(0.05)	[0.54–0.62]	0.62	(0.05)	[0.59–0.65]	0.001
	Width	0.14	(0.03)	[0.12–0.17]	0.14	(0.04)	[0.12–0.17]	0.702
Step 3	Average Forward Velocity (m.s.^-1^)	1.26	(0.13)	[1.16–1.35]	1.20	(0.12)	[1.12–1.29]	0.022
	Time (s)	0.55	(0.05)	[0.52–0.58]	0.57	(0.04)	[0.54–0.61]	0.011
	Width	0.13	(0.03)	[0.11–0.15]	0.12	(0.03)	[0.09–0.14]	0.003

## Consistent parameters between-tasks

Peak BCoM momentum during rising was greater in STW in the medio-lateral (toward the stance-limb) [*d* = 5.073, p<0.001] and AP (anterior) directions [*d* = 1.667, *p* = 0.001], but there was no difference vertically (Table 6). Despite seat-off occurring earlier in STW (Fig 2), there was no significant difference in flexion-momentum phase-time (movement-onset to seat-off). In contrast, transition phase-time (seat-off to GI-onset) [*d* = 3.362, *p*<0.001] were both significantly shorter in STW compared to STSW [*d* = 3.362, *p*<0.001]. In addition, maximum CoP-xCoM distances were greater during step1 [*d* = 1.558, *p* = 0.001] and 2 [*d* = 0.961, *p* = 0.014] in STW, but not during step3 (Table 6).

**Table 6:** Mean (SD) [95%CI] comparison for peak BCoM momentum, phase-time durations, and maximum CoP-xCoM distance during steps 1–3 between STW and STSW.

Movement Parameter		STW			STSW			p
		Mean	(SD)	[95%CI]	Mean	(SD)	[95%CI]	
Peak BCoM Momentum During Rising (kg.m.s^-1^)	Medio-Lateral	13.70	(2.65)	[11.81–15.60]	2.27	(0.85)	[1.66–2.88]	<0.001
	Anterior	39.61	(7.49)	[34.25–44.97]	34.00	(5.21)	[30.27–37.73]	0.001
	Vertical	42.45	(9.68)	[35.52–49.37]	40.16	(12.29)	[31.36–48.95]	0.338
Phase-Time Duration (s)	Flexion Momentum	0.61	(0.12)	[0.52–0.70]	0.65	(0.15)	[0.54–0.76]	0.088
	Transition	0.10	(0.03)	[0.07–0,12]	1.54	(0.44)	[1.23–1.85]	<0.001
Max CoP-xCoM Distance (m)	Step 1	0.55	(0.08)	[0.49–0.61]	0.50	(0.06)	[0.46–0.55]	0.001
	Step 2	0.61	(0.08)	[0.55–0.66]	0.58	(0.06)	[0.54–0.63]	0.014
	Step 3	0.67	(0.07)	[0.62–0.72]	0.66	(0.06)	[0.61–0.71]	0.168

**Fig 3:**
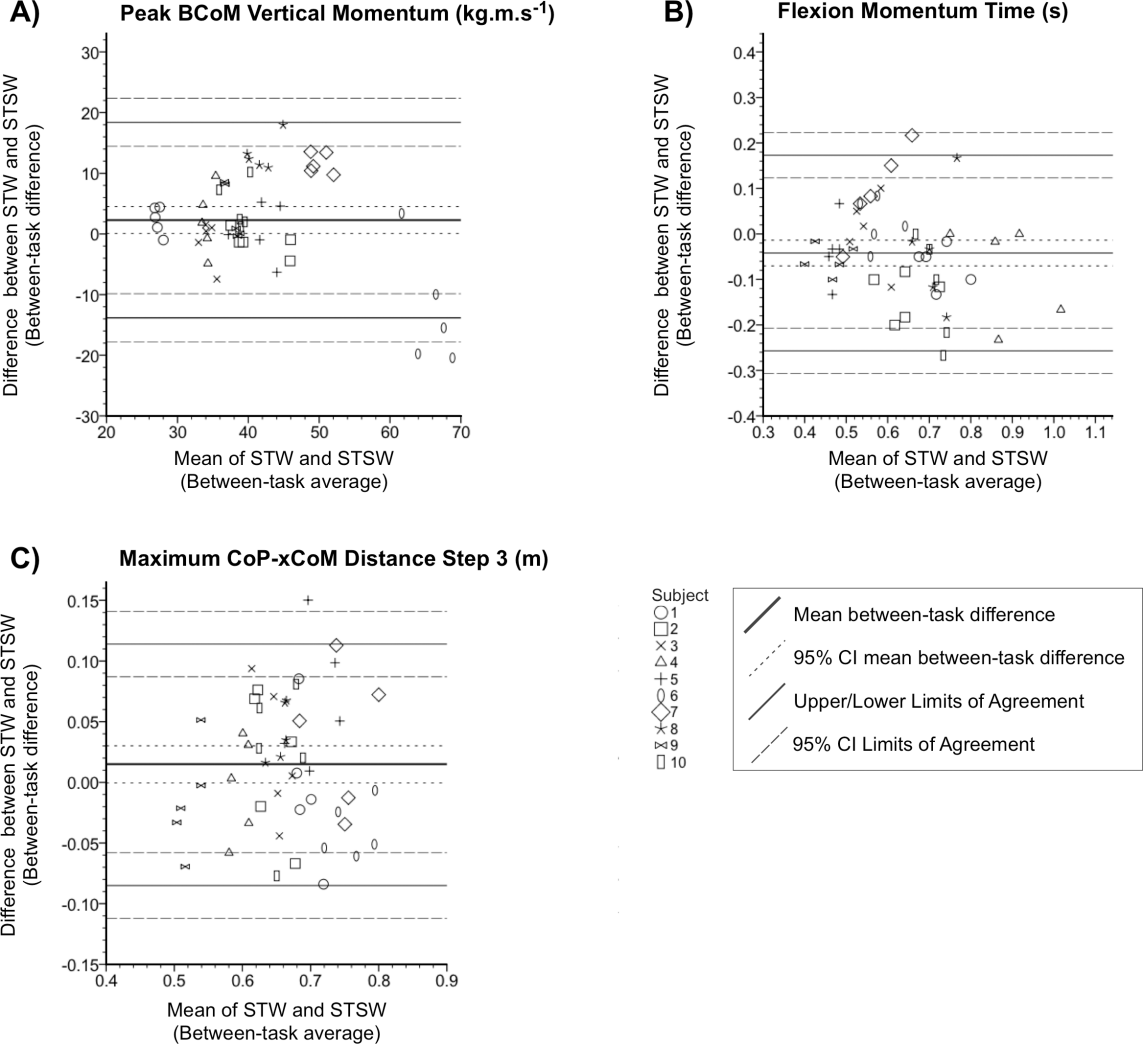
Bland and Altman plots. The differences (vertical axes) versus averages (horizontal axes) of STW and STSW for the three consistent movement parameters are shown; A) BCoM Vertical Momentum; B) Flexion-momentum time; C) Max CoP-xCoM Distance at Step3. Solid thick lines represent the mean between-task difference; short dashed lines represent the 95% confidence interval (CI) of the mean between-task difference. Lighter solid lines represent the limits of agreement (LOA), long dashed lines represent their 95% CIs.

Bland-Altman analyses revealed no statistically significant deviation of the mean between-task difference from zero in step3 CoP-xCoM max-distance [*t*(49) = 1.901, *p* = 0.063]. However, between-task difference for BCoM vertical momentum (2.287±1.137kg.m.s^-1^) and flexion-momentum time (0.102s (±0.014)) significantly deviated from zero [*t*(49) = 2.013, *p* = 0.050; *t*(49) = -2.915, *p* = 0.005 respectively] indicating systematic bias with larger BCoM vertical momentum and faster flexion-momentum time seen in STW (Fig 3). The between-task mean did not statistically predict the between-task difference in flexion-momentum time [F(1,48) = 3.507, *p* = 0.067; R^2^ = 0.068 ] or step3 CoP-xCoM max-distance [F(1,48) = 0.168, *p* = 0.684; R^2^ = 0.003]. It did however predict the between-task difference in BCoM vertical momentum [F(1,48) = 6.720, *p* = 0.013; R^2^ = 0.123].

STW ICC2,1 [95%CI] for BCoM vertical momentum was 0.928 [0.836–0.979], flexion-momentum time 0.753 [0.526–0.918], and step3 CoP-xCoM max-distance 0.812 [0.620–0.940]. While step3 CoP-xCoM max distance SEM was modest (0.039m), there were larger SEMs for peak BCoM vertical momentum (4.807kg.m.s-1) and flexion-momentum time (0.076s). This pattern was repeated with step3 CoP-xCoM max distance MDC (%MDC) being small [0.108m (16.2%)], whereas peak BCoM vertical momentum [13.323kg.m.s-1 (32.3%)] and flexion-momentum time [0.210s (33.4%)] were larger.

## Discussion

### Main findings

STW was characterised by greater vertical GRFs, forward momentum, fluidity, positional instability, and BCoM forward velocity compared to STSW. In contrast, peak vertical momentum, flexion-momentum time, and step3 positional stability remained consistent between-tasks, despite the imposition of a self-determined pause upon standing. These parameters yielded acceptable intra-subject reliability, with step3 stability demonstrating little systematic or proportional bias and low MDC in our small sample.

### Between-task discrimination

STW movement was executed rapidly (transition phase 0.10s±0.01s) and fluidly (22% mean HI) in a manner comparable to recent studies (0.14±0.03s [5]; 21%) [2]. In contrast, STSW was executed slowly (1.54±0.14s transition phase-time) and hesitantly (95% HI) as transition was delayed due to the imposition of a self-determined pause, and fluidity was constrained. However, our STSW pause duration (upright to GI-onset) was substantially shorter than the stabilisation phase favoured by healthy individuals (6.9±0.54s) after STS [22]. Therefore, future STSW studies should investigate the effect of the introduction of pauses of at least 7s in order to ensure stabilisation is achieved upon standing prior to GI.

Pausing once upright in STSW represents a movement control challenge reflected in a greater BCoM braking force (posterior GRF) (compared to STW) required to arrest forward propulsion, similar to that observed in STS [7, 23]. However, not pausing during STW was associated with greater forward BCoM momentum and GI occurring *during* rising. As a result GI was merged with STS around seat-off rather than GI occurring *after* upright is reached in STSW [10, 11]. As such, the instability associated with this rapid and fluid merging in STW represents the more significant motor control challenge [2, 5, 15].

Horizontal distance between CoP and BCoM is frequently used as an index of positional stability [24]. However, in this study the extrapolated whole-body-CoM (xCoM) was determined to account for the relative velocity of the BCoM [25], which has been shown to be more sensitive to dynamic instability [26]. We observed greater CoP-xCoM distances at seat-off, at both the onset and end (TO1) of GI, and at upright in STW–consistent with the exaggerated instability associated with the merging of rising and GI. While we failed to observe any between-task difference in response or flexion-momentum phase-times, greater peak forward BCoM momentums (commensurate with velocity) were evident in STW. Therefore, seat-off in STW represents the limit of equivalent BCoM forward velocity and is a key event after which positional instability is a function of rising and GI merging. The strategies adopted by individuals with pathology to control the greater instability in STW, and their relative effectiveness, remains however, to be determined.

### Consistent parameters across rise-to-walk performance

Peak BCoM vertical momentum during rising was the one of three parameters observed to consistent across STW and STSW. This was unexpected as shorter rise-time in STW is associated with greater average vertical BCoM velocity. Higher instantaneous peak velocities have been reported in STW compared to STS [7], when participants were required to adopt a constrained (hands-crossed-on-chest) arm position. Arm constraint is often used to minimise potential inter-subject differences [4, 6, 11] but is an approach that limits potentiation of leg extension forces [27], increases seat-off vertical force in STS [28], and ultimately is inconsistent with normal movement in the home and outside. With arms unconstrained, ankle joint variability is reduced, and the BCoM adopts a more forward position at seat-off [27]. It is possible therefore that BCoM peak vertical momentum was consistent between-tasks simply because the arms acted ‘naturally’ in positioning the BCoM to facilitate the efficiency of rising [29].

While there was acceptable intra-subject reliability in peak BCoM vertical momentum, flexion-momentum time, and 3^rd^ step stability, the MDC for BCoM vertical momentum was sizable meaning a difference between two measurements would need to exceed 13.3kg.m.s^-1^ to be 95% confident that it was not attributable to chance. In addition, Bland-Altman analyses yielded both systematic and proportional agreement bias between BCoM vertical momentum in STW and STSW suggesting that between-task agreement should be interpreted with caution from these results based on our small sample.

Mean flexion-momentum phase-time (~0.6s) was also consistent in our study between STW and STSW. Consistency has been observed previously between STS and STW, although phase-time was consistently longer (~0.8s) [7], which is probably explained by alternative movement-onset event characterisation. Another STS study using similar event characterisation to that we adopted observed comparable flexion-momentum phase-times (0.63s) [30]. Yet, compared to our study, their participants were taller, arms were constrained, and seat-height was lower. This suggests flexion-momentum time in healthy participants during rising is consistent irrespective of rising task, seat-height [13], and is not significantly affected by arm-use condition [28].

As flexion-momentum phase-time MDC was large, a difference between two measurements would need to exceed 0.21s (33.4%) to be statistically significant. In addition, although Bland-Altman analyses yielded no proportional agreement bias, there was a systematic bias between STW and STSW. This means the between-task agreement of this parameter should also be made with caution from these results based on our small sample.

Average BCoM forward velocity was greater by a clinically meaningful difference (0.1m.s^-1^) [31] during each step in STW. Furthermore, greater maximum positional instability was observed during steps1 and 2, but not step3 where stability converged, independent of velocity. Step3 maximum CoP-xCoM distance MDC was relatively low meaning a difference between two measurements would only need to exceed 0.11m (16.2%) to be statistically significant. In addition, because BCoM forward velocity yielded no systematic nor proportional agreement bias, between-task consistency was good.

In conclusion, while pausing in STSW requires greater AP braking force, merging of rising and GI in STW (around seat-off) is more challenging to control resulting in larger CoP-xCoM distances in STW. Whilst step3 maximum positional stability was the only consistent parameter yielding favourable agreement in our small cohort, we nonetheless observed two others (peak BCoM vertical momentum and flexion-momentum time) in healthy participants that remained consistent and reliable across normal rising-to-walk performance independent of self-selected pause. Our findings have implications for rehabilitation practice because these parameters are candidates with which to monitor change in transitional gait function following pathology by virtue of their consistency in health. Future studies should apply typical transition phase durations observed in pathology to larger groups of healthy subjects and determine whether any of these 3 parameters remain consistent during unconstrained (natural) rising-to-walk thereby enhancing their capability to monitor gait rehabilitation.
